# Experiences of family caregivers 3-months after stroke: results of the prospective trans-regional network for stroke intervention with telemedicine registry (TRANSIT-Stroke)

**DOI:** 10.1186/s12877-022-02919-6

**Published:** 2022-03-19

**Authors:** Steffi Jírů-Hillmann, Katharina M. A. Gabriel, Michael Schuler, Silke Wiedmann, Johannes Mühler, Klaus Dötter, Hassan Soda, Alexandra Rascher, Sonka Benesch, Peter Kraft, Mathias Pfau, Joachim Stenzel, Karin von Nippold, Mohamed Benghebrid, Kerstin Schulte, Ralf Meinck, Jens Volkmann, Karl Georg Haeusler, Peter U. Heuschmann

**Affiliations:** 1 Institute of Clinical Epidemiology and Biometry, University of Würzburg, Josef-Schneider-Str. 2/ D7, 97080 Würzburg, Germany; 2 Department of Applied Health Sciences, University of Applied Sciences, Bochum, Germany; 3 Charité – Universitätsmedizin Berlin, corporate member of Freie Universität Berlin and Humboldt Universität zu Berlin, Berlin, Germany; 4 Neurology, Leopoldina Hospital Schweinfurt, Schweinfurt, Germany; 5 Neurology, Clinical Centre Rhön, Bad Neustadt/Saale, Germany; 6 Neurology, Clinical Centre Aschaffenburg-Alzenau, Aschaffenburg, Germany; 7 Neurology, Clinical Centre Main-Spessart, Lohr, Germany; 8 Department of Neurology, University Hospital Würzburg, Würzburg, Germany; 9 Neurology, Clinical Centre Würzburg Mitte, site Juliusspital, Würzburg, Germany; 10 Internal Medicine, Clinical Centre Main, Ochsenfurt, Germany; 11 Cardiology, Clincal Centre Helios Frankenwald, Kronach, Germany; 12 Neurology, Clinical Centre Helios Erlenbach, Erlenbach, Germany; 13 Internal Medicine and Neurology, Rotkreuzklinik Wertheim, Wertheim, Germany; 14 Internal Medicine, Clinical Centre Franz von Prümmer, Bad Brückenau, Germany; 15 Clinical Trial Center, University Hospital Würzburg, Würzburg, Germany

**Keywords:** Family caregiver, Informal care, Stroke, Stroke care, Telemedicine network

## Abstract

**Background:**

Long-term support of stroke patients living at home is often delivered by family caregivers (FC). We identified characteristics of stroke patients being associated with receiving care by a FC 3-months (3 M) after stroke, assessed positive and negative experiences and individual burden of FC caring for stroke patients and determined factors associated with caregiving experiences and burden of FC 3 M after stroke.

**Methods:**

Data were collected within TRANSIT-Stroke, a regional telemedical stroke-network comprising 12 hospitals in Germany. Patients with stroke/TIA providing informed consent were followed up 3 M after the index event. The postal patient-questionnaire was accompanied by an anonymous questionnaire for FC comprising information on positive and negative experiences of FC as well as on burden of caregiving operationalized by the *Caregiver Reaction Assessment* and a *self-rated burden-scale*, respectively. Multivariable logistic and linear regression analyses were performed.

**Results:**

Between 01/2016 and 06/2019, 3532 patients provided baseline and 3 M-follow-up- data and 1044 FC responded to questionnaires regarding positive and negative caregiving experiences and caregiving burden. 74.4% of FC were older than 55 years, 70.1% were women and 67.5% were spouses. Older age, diabetes and lower Barthel-Index in patients were significantly associated with a higher probability of receiving care by a FC at 3 M. Positive experiences of FC comprised the importance (81.5%) and the privilege (70.0%) of caring for their relative; negative experiences of FC included financial difficulties associated with caregiving (20.4%). Median overall self-rated burden was 30 (IQR: 0–50; range 0–100). Older age of stroke patients was associated with a lower caregiver burden, whereas younger age of FC led to higher burden. More than half of the stroke patients in whom a FC questionnaire was completed did self-report that they are not being cared by a FC. This stroke patient group tended to be younger, more often male with less severe stroke and less comorbidities who lived more often with a partner.

**Conclusions:**

The majority of caregivers wanted to care for their relatives but experienced burden at the same time. Elderly patients, patients with a lower Barthel Index at discharge and diabetes are at higher risk of needing care by a family caregiver.

**Trial registration:**

The study was registered at “German Clinical Trial Register”: DRKS00011696. https://www.drks.de/drks_web/navigate.do?navigationId=trial.HTML&TRIAL_ID=DRKS00011696

**Supplementary Information:**

The online version contains supplementary material available at 10.1186/s12877-022-02919-6.

## Introduction

Stroke is a major cause of long-term disability with most stroke patients returning home after acute hospital care [1–4]. Long-term support for stroke patients at home is delivered by formal and informal caregivers [5–7]. Family caregiver (FC) are the primary support for stroke survivors [6, 8, 9]. However, caregivers are often not prepared for their role and adequate information regarding different aspects of caregiving is not available [7]. Caring for a disabled relative with stroke is often described as straining for the caregiver itself [10–12] and might also be associated with negative experiences like the need to disrupt caregivers’ work [13]. In addition, mental consequences such as depressive symptoms, lower life satisfaction, and restraints in mental health quality of life might be more frequently observed in caregivers of stroke patients compared to demographically similar controls [14]. However, some caregivers also report positive aspects of caregiving such as companionship, fulfillment, and enjoyment [15]. Individual factors of patients and caregiver affecting caregiver strain are still not fully understood [12] and it remains unclear to what extent individual factors were associated with caregiver burden as well as positive or negative experiences [16, 17]. Additionally, data regarding the situation of FC of stroke patients in Germany are scarce.

Therefore, we investigated demographical characteristics and personal circumstances of FC of stroke patients and identified demographic and clinical determinants of stroke patients being associated with receiving care by a FC three months after stroke using different scenarios of family care. We assessed positive and negative experiences as well as individual burden of FC caring for stroke patients and determined factors associated with caregiving experiences and burden of FC three months after stroke within a large cohort of prospectively enrolled stroke patients in Germany.

## Methods

### Setting

Data were collected within the Trans-regional Network for Stroke Intervention with Telemedicine (TRANSIT-Stroke). TRANSIT-Stroke is a telemedical stroke network comprising 12 hospitals with different levels of stroke care in a predominantly rural area in Northwest Bavaria (Germany). Level-III-hospitals (*n* = 4) run a supra-regional stroke unit (SU) certified according to the German Stroke Society (DSG) criteria. Level-II-hospitals (*n* = 2) run a certified regional SU, and level-I-hospitals (*n* = 6) have no certified SU but run an intensive or intermediate care facility. The region and structure of the TRANSIT-Stroke network has been described in detail elsewhere [18].

### Study population

Between October 2014 and June 2019, patients with stroke or TIA (ICD 10: I61, I63, I64, G45) treated within the TRANSIT-network were eligible and, thus, invited for a postal or telephone follow-up 3 months after stroke. From January 2016 onwards, information on family caregiver (FC) experiences was also documented in the follow-up. Therefore, data collected between 2016 and 2019 were included in the current analysis. The postal patient questionnaire was accompanied by a separate anonymous questionnaire for FC including information on positive and negative experiences of FC as well as on burden of caregiving. Questionnaires of the FC were included in the analysis if the FC was a family member and if the stroke patient was not institutionalized in a care home.

### Data collection

Baseline data of stroke or TIA patients were collected during hospital stay by trained staff members and were documented within the Bavaria stroke register, a member of the German Stroke Registers Study Group (ADSR) [19]. Documented information included diagnostics, treatment, co-morbidities, risk factors, complications and discharge information. For the 3-month follow-up, the following algorithm was used: First, an initial questionnaire was mailed. Non-responders were reminded up to 3 times by phone and one time by mail after 3 and 5 weeks. In a last attempt, non-responders were contacted again up to 3 times by phone, one time by mail and a last attempt by phone.

For stroke patients, information at 3 months-FU was collected on vital status, stroke recurrence, activities of daily living (Barthel Index), degree of disability and dependency (modified Rankin Scale), current living situation, rehabilitation services after hospital discharge, patient satisfaction (The German version of the satisfaction with stroke care questionnaire [SASC]) [20] and depressive symptoms (Patient Health Questionnaire [PHQ-9]) [21].

For FCs, data was collected on sociodemographic characteristics, such as age in categories (18–35, > 35–55, > 55–75, > 75), and sex, as well as their marital status (married, widowed, divorced, single) and their employment status (full-time/part-time, retired, unemployed, housewife/househusband, other). In addition, their relationship to the stroke patient (spouse/partner, daughter/son, daughter−/son in law, sibling), other (aunt/uncle, cousin, nephew, niece), was requested, the average number hours of provided care per week, as well as whether they cared for the patient before or since stroke.

Two instruments for assessing caregiver’s positive and negative experiences and overall individual burden were used. The *Caregiver Reaction Assessment (CRA)* was developed in 1992 by Given et al. [22] and provides positive and negative experiences of caregiving. In 2012, Stephan et al. developed a validated German version of the CRA (G-CRA) [23]. Details of the questionnaire, including every question, are provided within the supplemental material. The G-CRA consists of 24 items and five domains. Four of them record negative polarity: impact on finances, lack of family support, impact on health, impact on daily schedule, while one of them records positive polarity: caregivers` self-esteem. Except for the domain “impact on daily schedule”, all domains contain reverse questions. Agreement is assessed on a 5-point-Likert-Scale (strongly agree; agree; neither agree nor disagree; disagree; strongly disagree) [23]. Reverse questions of the G-CRA were recoded and summed up to each domain. In addition, the one-item *self-rated burden scale (SRB)* [0–100] was used to indicate overall burden of how burdensome caring is at the moment (0: caring is not straining at all and 100: caring is much too straining) [24].

### Statistical analysis

Descriptive analysis for demographic and clinical characteristics were presented as frequencies (proportions) or means (standard deviation). Care status was descriptively compared using appropriate tests, such as Chi^2^-test or t-test. Univariable and multivariable logistic regression analyses were calculated to identify determinants to receive family care. Multivariable analyses were adjusted for age, sex, stroke subtype, Barthel Index in categories (100: independent, 60–95: independent with support, < 60: dependent) [25] at discharge, comorbidities (previous stroke, atrial fibrillation, diabetes, and hypertension) and SU-level. To identify patient and FC characteristics associated with positive and negative caregiving experiences (domains of the G-CRA) and self-rated burden (SRB), multiple linear regression analyses were performed in the subgroup of the caregivers with a completed G-CRA. Multivariable analyses were adjusted for patient characteristics (age and sex, Barthel Index at 3 months, depressive symptoms [PHQ-9, in categories: 0: none, 0–4: minimal, 5–9: mild, 10–14: moderate, 15–19: moderately severe, 20–27: severe] [21] and ambulant nursing service) and caregiver characteristics (age in categories, sex, and hours of care per week). Backward selection was used to exclude insignificant variables from the model. All tests were two-tailed and statistical significance was determined at an alpha level of 0.05. Statistical analyses were performed with SAS Software, version 9.4 (SAS Institute, Inc., Cary, NC).

### Ethics

The data collection of the TRANSIT-Stroke network has been approved by the Ethic Committee of the University of Würzburg (54/14) and was registered in the German Registry for Clinical Studies (DRKS) (registration number: 11696; registration date: 11/08/2017). All patients or their legal representatives provided written informed consent to participate. Data collection of the FC was anonymous and, therefore, no dedicated written informed consent was collected. Data collection procedures were approved by data protection officer.

## Results

Between January 2016 and June 2019, at total of 19,820 cases of stroke or TIA were treated within the network. Of those, 5731 (28.9%) patients gave written informed consent to participate in the TRANSIT-Stroke registry. Overall, 3654 (63.8%) stroke patients provided data at baseline and 3-months follow-up; of those, 122 patients lived in a care home and were, therefore, excluded from the present analyses. Of 3532 stroke patients included in the dataset, 1044 FCs filled out the separate questionnaire regarding positive and negative experiences and a self-rated overall burden. (Fig. I supplemental).

### Demographic characteristics and personal circumstances of FCs

Women were the main FC (70.1%) and 74.4% of FCs were older than 55 years. 67.5% were a spouse or a partner of the stroke patient. The median hours of provided care per week was 14 (IQR: 5–28). Of all 1044 questionnaires answered by the FC, 426 patients reported being cared for by a FC and 618 reported not to receive care by a FC. (Fig. I supplemental) At 3-months follow-up, patients who reported not to receive care by a FC were predominantly taken care for by a spouse or a partner compared to patients who reported to receive care by a FC (*p* < .0001) and received less hours of care per week (p < .0001). (Table 1).

**Table 1:** Demographic characteristics and personal circumstances of FCs

		Patient self-reported to receive care by a FC		
	All	Yes	No	*P* Value
Number	*N* = 1044	*N* = 426	*N* = 618	
Age categories, %				0.1147
18–35	2.9	1.5	3.8	
> 35–55	22.8	22.1	23.2	
> 55–75	51.4	51.6	51.3	
> 75	23.0	24.8	21.6	
Women, %	70.1	71.8	68.9	0.3292
Relationship to stroke patient, %				<.0001
Spouse/Partner/Mate	67.5	56.9	75.1	
Daughter/Son / Daughter−/Son in law	27.3	36.6	20.7	
Sibling	2.5	2.7	2.4	
Other	2.7	3.8	1.8	
Hours of care per week, h				<.0001
Median (IQR)	14 (5–28)	23 (14–40)	7 (2–15)	
Caring for the relative, %				0.5694
Before stroke	45.4	46.4	44.5	
Since stroke	54.6	53.6	55.5	
Marital status, %				0.2524
Married	88.4	86.9	89.5	
Widowed	1.9	1.7	2.1	
Divorced	3.1	4.3	2.2	
Single	6.6	7.2	6.2	
Employment status, %				0.5039
Full-time/Part-time position	34.0	32.1	35.4	
Retired	46.3	45.9	46.6	
Unemployed	1.8	1.7	1.9	
Housewife/Househusband	13.9	15.9	12.4	
Other	4.0	4.4	3.7	

### Demographic and clinical characteristics of stroke patients associated with receiving care by a FC by different scenarios of family caregiving

The association between demographic and clinical characteristics of stroke patients with receiving care was investigated for different scenarios of family caregiving due to the divergent responses in the questionnaire. Family care was defined as follows: 1) care self-reported by FC; 2) care self-reported by the patient; 3) any type of care reported by the patient or the FC. In all these scenarios, the following patient characteristics were significantly associated with a higher probability to receive care by a FC in univariable (data not shown) and multivariable logistic regression analysis in Table 2: older age, a lower Barthel Index at discharge as well as having diabetes. Patients treated in hospitals with a Stroke Unit had a lower probability to receive care by a FC.(Table 2).

**Table 2:** Demographic and clinical characteristics of stroke patients associated with receiving care by a FC by different scenarios of family caregiving

	Care self-reported by FC (n = 1044)		Patients self-reported to receive care by a FC (*n* = 604)		Any type of care indicated by patient and / or FC (*n* = 1222)	
Multivariable analysis						
	OR (95% CI)	P Value	OR (95% CI)	P Value	OR (95% CI)	P Value
Age categories						
< 65	1	<.0001	1	<.0001	1	<.0001
65–74	1.35 (1.09–1.68)		1.82 (1.30–2.54)		1.36 (1.10–1.69)	
75–84	1.60 (1.30–1.97)		3.04 (2.22–4.16)		1.93 (1.57–2.37)	
> = 85	2.36 (1.73–3.23)		7.42 (4.99–11.03)		3.40 (2.48–4.66)	
Sex, women	0.74 (0.63–0.88)	0.0003	0.89 (0.72–1.10)	0.2781	0.76 (0.64–0.89)	0.0005
Stroke subtype						
Ischemic Stroke	1	0.0501	1	0.3908	1	0.0031
TIA	0.78 (0.64–0.94)		0.83 (0.63–1.08)		0.72 (0.59–0.87)	
Hemorrhagic Stroke	1.20 (0.69–2.09)		1.30 (0.63–2.68)		1.39 (0.80–2.43)	
Unknown	1.93 (0.32–11.84)		2.06 (0.20–21.02)		1.52 (0.24–9.55)	
Barthel Index, discharge						
100	1	<.0001	1	<.0001	1	<.0001
> = 60 - < =95	2.15 (1.80–2.58)		3.63 (2.90–4.54)		2.40 (2.01–2.87)	
< 60	3.66 (2.74–4.89)		11.96 (8.65–16.54)		5.52 (4.05–7.53)	
Comorbidities						
Previous stroke	1.12 (0.93–1.34)	0.2461	1.55 (1.23–1.94)	0.0002	1.27 (1.06–1.52)	0.0114
Atrial fibrillation	1.05 (0.87–1.27)	0.6243	1.09 (0.86–1.38)	0.4821	1.01 (0.84–1.22)	0.9154
Diabetes	1.29 (1.07–1.54)	0.0065	1.81 (1.45–2.26)	<.0001	1.38 (1.15–1.65)	0.0005
Hypertension	0.95 (0.76–1.19)	0.6358	1.05 (0.74–1.48)	0.7831	0.97 (0.78–1.21)	0.7753
Hospital level						
Hospital without SU*	1	0.0209	1	0.0542	1	0.0096
Hospital with regional SU*	0.67 (0.50–0.90)		0.63 (0.43–0.92)		0.67 (0.50–0.89)	
Hospital with supra-regional SU*	0.73 (0.57–0.94)		0.71 (0.52–0.99)		0.69 (0.54–0.89)	

* SU (Stroke Unit)

Descriptive demographic and clinical characteristics of stroke patients at baseline and 3-months of FU are shown in supplemental Table I. The results of the 3 scenarios of family care show that patients who reported being cared for by a FC were older (*p* < .0001), tended to be more often male (care self-reported by FC: *p* = 0.0545), had a more severe stroke (*p* < .0001), were more dependent on admission (*p* < .0001) and at discharge (*p* < .0001), and had more comorbidities (diabetes [*p* < .0001], atrial fibrillation [care self-reported by FC: *p* = 0.0002; care self-reported by the patient/ any type of care: *p* < .0001], hypertension [care self-reported by FC: *p* = 0.0007; care self-reported by the patient/ any type of care: *p* < .0001], previous stroke [p < .0001]). After 3 months, those stroke patients were more dependent (p < .0001), had more depressive symptoms according to the PHQ-9 (p < .0001), and received more frequently care by an ambulant nursing services (p < .0001) compared to patients reporting not to receive care. (Supplemental Table I) This also applies to those stroke patients of whom a FC answered the questionnaire (1044 FC questionnaires; of those 426 patients answered to receive care by a FC and 618 answered not to receive care by a FC) (data not shown).

### Positive and negative experiences and overall self-rated burden of FC

Table II (supplemental) shows the positive and negative experiences of the FC. About one fifth (20.4%) agreed that it is difficult to pay for the patient’s health needs and services. 44.1% of the FC reported that the family works together at caring for the relative. 23.4% did not have enough strength to care and 22.5% were tired all the time since taking care of their relative. 36.8% eliminated things from their daily schedule and nearly one third (29.1%) agreed, that constant interruptions make it difficult to find time for relaxation. 81.5% of FC agreed that caring is important to them. Also the majority of FC agreed to want to care (73.0%) or felt privileged (70.0%) to care for their relative (Table II supplement). The median self-rated burden was 30 (IQR: 0–50).

### Patient and family caregiver characteristics associated with positive and negative caregiving experiences and self-rated burden of FC

Using backward selection procedure, caring before stroke and employment status of FC were statistically not significant and, therefore, removed from the model. Depressive symptoms in patients at 3 months were positively associated with the overall self-rated burden (*p* < .0001) all domains of the positive and negative experiences (impact on finances, *p* = 0.0001; lack of family support, *p* = 0.0020; impact on health, *p* = 0.0019; impact on daily-schedule, *p* < .0001) except the caregivers’ self-esteem domain (*p* = 0.3348) with positive polarity. Patients’ Barthel Index at 3 months was negatively associated with impact on finances (*p* = 0.0098), impact on health (0.0353), impact on daily-schedule (*p* = 0.0068), and the self-rated burden scale (p < .0001). Of the individual items of the Barthel Index, feeding, bathing and bladder were negatively associated with the self-rated burden scale. (Table III). The utilization of an ambulant nursing service showed no significant impact on the experience of the caregiver.

Higher patient age was negatively associated with the impact on finances (*p* = 0.0023) and positively with the self-rated burden scale (*p* = 0.0020). Hours of care per week were positively associated with impact on health (*p* = 0.0104), the impact on daily schedule (*p* < .0001), and the self-rated burden (*p* = 0.0038). (Table 3).

**Table 3:** Patient and family caregiver characteristics associated with positive and negative caregiving experiences and self-rated burden of FC

Subscale	*β* *	95% CI for *β*	P value
Impact on finances			
Patient characteristics			
Age patient	−0.049	−0.080 to − 0.018	0.0023
Sex (Man)	−0.457	−1.249 to 0.335	0.2566
Patient 3 months			
Barthel Index	−0.028	−0.049 to − 0.007	0.0098
Depressive Symptoms	0.123	0.060 to 0.186	0.0001
Ambulant nursing service	0.052	−1.044 to 1.148	0.9258
Caregiver characteristics			
Age	−0.393	−0.857 to 0.071	0.0966
Sex (Man)	−0.591	−1.396 to 0.214	0.1497
Hours of care /week	0.004	−0.005 to 0.013	0.4128
R^2^ = 0.1618			
Lack of family support			
Patient characteristics			
Age patient	0.043	−0.002 to 0.088	0.0592
Sex (Man)	−0.276	−1.422 to 0.871	0.6365
Patient 3 months			
Barthel Index	−0.003	−0.034 to 0.027	0.8246
Depressive Symptoms	0.142	0.052 to 0.231	0.0020
Ambulant nursing service	0.770	−0.801 to 2.342	0.3355
Caregiver characteristics			
Age	−0.676	−1.339 to − 0.013	0.0459
Sex (Man)	0.280	−0.891 to 1.451	0.6378
Hours of care /week	0.001	−0.012 to 0.014	0.8903
R^2^ = 0.0807			
Impact on health			
Patient characteristics			
Age patient	−0.006	−0.041 to 0.030	0.7585
Sex (Man)	−0.629	−1.530 to 0.272	0.1706
Patient 3 months			
Barthel Index	−0.026	− 0.050 to − 0.002	0.0353
Depressive Symptoms	0.1128	0.042 to 0.184	0.0019
Ambulant nursing service	0.924	−0.324 to 2.172	0.1462
Caregiver characteristics			
Age	−0.112	− 0.638 to 0.414	0.6751
Sex (Man)	0.597	−0.328 to 1.521	0.2052
Hours of care /week	0.014	0.003 to 0.025	0.0104
R^2^ = 0.1915			
Impact on daily schedule			
Patient characteristics			
Age patient	0.044	−0.001 to 0.089	0.0572
Sex (Man)	−0.969	−2.153 to 0.216	0.1086
Patient 3 months			
Barthel Index	−0.043	− 0.074 to − 0.012	0.0068
Depressive Symptoms	0.324	0.232 to 0.415	<.0001
Ambulant nursing service	1.066	−0.526 to 2.657	0.1885
Caregiver characteristics			
Age	−0.157	−0.845 to 0.531	0.6544
Sex (Man)	−0.102	−1.311 to 1.108	0.8689
Hours of care /week	0.028	0.015 to 0.041	<.0001
R^2^ = 0.4029			
Caregivers self-esteem			
Patient characteristics			
Age patient	0.013	−0.047 to 0.072	0.6797
Sex (Man)	0.490	−1.058 to 2.038	0.5338
Patient 3 months			
Barthel Index	−0.009	− 0.051 to 0.034	0.6789
Depressive Symptoms	−0.060	−0.181 to 0.062	0.3348
Ambulant nursing service	−1.479	−3.634 to 0.675	0.1776
Caregiver characteristics			
Age	−0.248	−1.139 to 0.644	0.5848
Sex (Man)	−0.297	−1.890 to 1.296	0.7140
Hours of care /week	0.018	−0.001 to 0.036	0.0585
R^2^ = 0.4562			
Self-rated burden			
Patient characteristics			
Age patient	0.379	0.140 to 0.618	0.0020
Sex (Man)	−3.519	−9.654 to 2.616	0.2600
Patient 3 months			
Barthel Index	−0.369	− 0.534 to − 0.205	<.0001
Depressive Symptoms	1.261	0.780 to 1.743	<.0001
Ambulant nursing service	7.628	−0.909 to 16.164	0.0797
Caregiver characteristics			
Age	−4.515	−8.106 to −0.924	0.0139
Sex (Man)	−1.628	−7.954 to 4.698	0.6129
Hours of care /week	0.105	0.034 to 0.176	0.0038
R^2^ = 0.3949			

* *β:* regression coefficient

## Discussion

Based on a large cohort recruited from clinical routine in Germany, we described demographic and clinical characteristics of family caregiver and the stroke patients receiving family care. We identified positive and negative experiences as well as individual burden of FCs. Furthermore, we were able to identify determinants associated with positive and negative caregiving experiences. Stroke patients being cared for by a FC three months after the event were older, had a lower Barthel Index at discharge and more often suffered from comorbidities. 74.4% of the FCs were older than 55 years, 70.1% were females and 67.5% were spouses or partners; the median duration of care per week was 14 h (IQR: 5–28). Median overall self-rated burden was 30 (0–50). Older patient age, depressive symptoms in patients as well as hours of care per week was associated with higher self-rated burden. Most FC want to care (73%) and feel privileged (70%) to care for the stroke patients. About a fifth of FC reported financial problems (20.4%) and being tired all the time (22.5%). Depressive symptoms in stroke patients, patients’ Barthel Index at 3-months and number of provided hours of care per week were associated with the most domains of the positive and negative experiences questionnaire and self-rated burden of FC.

In our study, demographic characteristics of FCs were comparable with previous data from cross-sectional, case-control and cohort studies, mainly from European countries. In accordance to our findings, in previous studies, the majority of caregivers were women [14–16, 26–30] and about 74% of FCs were aged 55 years and older [14, 15, 27, 28]. In addition, also comparable to previous studies, spouses and partners were the main care provider (67.5%) [26, 28, 29]. There are a few publications studying determinants to receive informal care [31]. However, different variables have been explored in previous studies, hampering direct comparisons with our results. In general, older age seems to be a predictor to receive informal care [32] as well as stroke severity at discharge and the individual’s health related quality of life [31]. This information might be helpful to identify patients at high risk of informal care and their potential FCs during the hospital discharge process and to provide them with dedicated information, e.g. on support programs for informal caregivers [33].

About a fifth of caregivers in our study reported financial strains associated with caregiving what seems to be a general problem being associated with caregiving. Within the caregivers’ self-esteem domain, FCs stated that they wanted to care for their relative and that caring for their relative was important to them [34, 35]. This domain consist of mainly positive formulated questions that might be easier to confirm than negative formulated ones [34]. Caring has an impact on the daily schedule of FCs. Hence, about two third had to eliminate things from their daily schedule and visited family and friends less often, as also described previously in informal and family caregivers of stroke patients [1, 36].

Depressive symptoms of patients were associated with higher caregiver burden for the self-rated burden and all domains of the positive and negative experiences, except the self-esteem domain with positive polarity. Within the literature, mental health problems of patients and caregivers are associated with increased caregiver burden [1, 37, 38]. A higher Barthel Index of stroke patients at 3 month was associated with lower self-rated-burden in caregivers and lower caregiver burden within the financial-domain, the daily-schedule domain and the impact on health domain of family caregivers. Comparable to these findings, a higher functional status was associated with lower caregiver burden in several publications [32, 39, 40]. More hours of care per week were found to be related to a higher burden regarding the impact on health domain, the impact on daily-schedule domain and the self-rated burden scale. Long hours of caregiving were a contributing factor regarding caregiver burden in previous studies [41].

A higher caregiver age was associated with lower self-rated burden. This was also found in recent literature [16], whereas the influence of caregivers’ age on experienced burden was inconclusive in a review of determinants of overburdening among informal carers [32]. However, this review included also other medical conditions such as dementia and solid tumors [32]. A Canadian study with 133 caregivers found that older caregivers had more self-esteem, more family support, and less financial problems in a comprehensive set of geriatric care services [42].

In our study, we were able to show for the first time a difference in perception whether stroke patients acknowledge that they are being cared by a FC. More than half of the stroke patients in whom a FC reported to provide care, self-reported that they are not being cared by a FC. This stroke patient group tended to be younger, more often male with less severe stroke and less comorbidities who lived more often with a partner and received less hours of care per week. A possible explanation of this observation might be that care is seen as “normal” by the care recipient, especially care by a female partner, whereas the actual caregiver considers it as family care. This result suggests that it is important to take also the individual perspective of family caregivers into account if stroke patients are evaluated regarding their individual care needs. Another possible explanation might be that FC exaggerate their role, and on the other hand stroke patients might have cognitive deficits that limit their perception of being cared by a FC. A group of stroke patients reported to receive care by a FC but no questionnaire from a FC was available. This might be because some FC did not label themselves as caregivers or did not have the time and energy to participate. Overall, due to the wide range of hours of care per week and the lack of standardization of the definition of care, we assume a considerable amount of heterogeneity regarding the types of care provided by the FC.

This study has several strengths. We were able to cover a defined geographical region and collected a large data set for a substantial number of patients from different levels of clinical routine. Additionally, we were able to include the perspective of patients and family caregivers on receiving care. However, there are also limitations that should be acknowledged. As informed consent was needed, patients in our registry tended to be younger than the average stroke patient. In addition, selection bias due to low response rates is a major limitation of our study. Reported data may not be representative and may not be applicable to whole Germany and on an international level. We had a relatively short follow-up period of three months after the index event and cannot make any statements about changes in the physical and mental health of FC. Additionally, long-term caregiver burden might differ. However, previous studies showed that caregiver burden remains relatively stable over time [12, 13]. Duration of care was not assessed on a daily basis which would have been more detailed. Due to data protection regulations, we collected caregiver information in an anonymous way, hampering the documentation of detailed personal information such as day of birth. We cannot provide the total number of patients who are actually cared for by a family caregiver. The number of questionnaires of FC might not be complete, as 178 stroke patients reported to receive care by a FC but no questionnaire was available from a FC. Explained variance differed in our model for each domain of the positive and negative experiences between 0.0807 (lack of family support) and 0.4562 (caregivers’ self-esteem) and 0.3949 for the self-rated burden. Therefore, we cannot rule out residual confounding in our analysis.

## Conclusion

Even though research on the topic of experiences and burden of family caregiver is going on for several years, there is still room to improve the situation of family caregiver. The majority of caregivers in our study wanted to care for their family member but were at risk of burden and health disadvantages at the same time. Elderly patients, patients with a lower Barthel Index at discharge as well as having diabetes are at higher risk of needing care by a family caregiver. Knowing these factors makes it possible to identify potential FC during hospital stay and provide them with information about existing support programs for FC, for instance. FC of stroke patients with depression might also be prioritized for support programs due to their higher level of experiences burden. FC eliminated things from their daily schedule like visiting family and friends less often. To relieve the burden on FC, the offer of day nursing services for stroke patients could be enhanced. Due to financial problems of family caregivers, long term care allowances could improve financial shortcomings of FC. Younger, male stroke patients, living with a spouse or partner, with less severe stroke seem to be often not aware of being cared by a family caregiver whereas the family caregiver considers it as actual care. Thus, the perspective of caregivers should also be taken into account if patients are evaluated for care needs.

## Supplementary Information

**Additional file 1.**

## Abbreviations

G-CRA: German Caregiver Reaction Assessment
FC: family caregiver
SU: stroke unit
SRB: Self-Rated Burden scale
TRANSIT-Stroke: Trans-regional Network for Stroke Intervention with Telemedicine
