# Supplementary material for: Experiences of family caregivers 3-months after stroke: results of the prospective trans-regional network for stroke intervention with telemedicine registry (TRANSIT-Stroke)
Source: BMC Geriatr. 2022 Mar 19;22:228. doi: 10.1186/s12877-022-02919-6 (PMC8934512; doi:10.1186/s12877-022-02919-6)

**Additional files**

File name: Additional file_caregiver_experiences_TRANSIT-Stroke.docx

Title of data: Table I Demographic and clinical patient characteristics at baseline and 3-months post stroke

Description of data: analysis of patient characteristics

Title of data: Table II Results of the Caregiver Reaction Assessment – positive and negative experiences of FC

Description of data: descriptive analysis of Caregiver Reaction Assessment questionnaire

Table III Patient characteristics including single items of the Barthel Index and family caregiver characteristics associated with self-rated burden of FC

Figure I Flow Chart

Description of data: Flow Chart of patients and family caregivers participated

SUPPLEMENTAL

Table I Demographic and clinical patient characteristics at baseline and 3-months post stroke

|  | All patients | Care self-reported by FC | | | Care self-reported by patient | | | Any type of care indicated by patient and/or FC | | |
| --- | --- | --- | --- | --- | --- | --- | --- | --- | --- | --- |
|  |  | Yes | No | P Value | Yes | No | P Value | Yes | No | P Value |
| **Baseline – acute phase** |  |  |  |  |  |  |  |  |  |  |
| Number, n | 3,532 | 1,044 | 2,488 |  | 604 | 2,928 |  | 1,222 | 2,310 |  |
| Age, y, Median (IQR) | 72 (62-79) | 75 (66-81) | 70 (60-78) | <.0001 | 78 (72-84) | 70 (60-78) | <.0001 | 76 (66-82) | 69 (59-78) | <.0001 |
| Women, % | 38.9 | 36.5 | 40.0 | 0.0545 | 42.1 | 38.3 | 0.0838 | 37.6 | 39.7 | 0.2251 |
| Stroke subtype, % |  |  |  | <.0001 |  |  | 0.0005 |  |  | <.0001 |
| Ischemic Stroke | 74.6 | 78.8 | 72.9 |  | 79.5 | 73.6 |  | 79.2 | 72.2 |  |
| Transient Ischemic Attack | 23.4 | 18.5 | 25.4 |  | 17.6 | 24.6 |  | 18.1 | 26.2 |  |
| Hemorrhagic Stroke | 1.8 | 2.4 | 1.6 |  | 2.8 | 1.6 |  | 2.5 | 1.5 |  |
| Unknown | 0.2 | 0.3 | 0.1 |  | 0.2 | 0.2 |  | 0.3 | 0.1 |  |
| NIHSS, admission |  |  |  | <.0001 |  |  | <.0001 |  |  | <.0001 |
| Mean (SD) | 2.9 (3.5) | 3.8 (4.1) | 3.8 (4.1) |  | 4.4 (3.0) | 2.6 (2.0) |  | 3.8 (4.1) | 2.4 (3.05) |  |
| Median (IQR) | 2 (1-4) | 3 (1-5) | 3 (1-5) |  | 3 (1-6) | 2 (0-3) |  | 3 (1-5) | 2 (0-3) |  |
| Barthel Index on admission, % |  |  |  | <.0001 |  |  | <.0001 |  |  | <.0001 |
| Independent | 48.6 | 37.0 | 53.4 |  | 21.7 | 54.1 |  | 34.8 | 55.8 |  |
| Independent with little help | 34.9 | 38.5 | 33.4 |  | 41.4 | 33.5 |  | 38.8 | 32.8 |  |
| Dependent | 16.5 | 24.5 | 13.2 |  | 36.9 | 12.4 |  | 26.4 | 11.3 |  |
| Barthel Index at discharge, % |  |  |  | <.0001 |  |  | <.0001 |  |  | <.0001 |
| Independent | 68.2 | 52.1 | 74.9 |  | 32.6 | 75.5 |  | 50.1 | 77.8 |  |
| Independent with little help | 23.2 | 33.5 | 18.9 |  | 42.9 | 19.1 |  | 34.3 | 17.3 |  |
| Dependent | 8.6 | 14.4 | 6.2 |  | 24.5 | 5.4 |  | 15.6 | 4.9 |  |
| Comorbidities, % |  |  |  |  |  |  |  |  |  |  |
| Diabetes | 22.2 | 27.1 | 20.1 | <.0001 | 34.5 | 19.6 | <.0001 | 27.7 | 19.2 | <.0001 |
| Atrial fibrillation † | 19.8 | 23.7 | 18.2 | 0.0002 | 28.8 | 18.0 | <.0001 | 24.0 | 17.6 | <.0001 |
| Hypertension | 82.8 | 86.1 | 81.4 | 0.0007 | 91.5 | 81.0 | <.0001 | 87.0 | 80.5 | <.0001 |
| Previous stroke | 21.1 | 25.2 | 19.4 | 0.0001 | 33.7 | 18.5 | <.0001 | 26.7 | 18.2 | <.0001 |
| Hospital level, % |  |  |  | 0.3182 |  |  | 0.2753 |  |  | 0.1112 |
| Hospital without SU ‡ | 12.5 | 13.8 | 12.0 |  | 14.4 | 12.1 |  | 14.1 | 11.7 |  |
| Hospital with regional SU ‡ | 19.1 | 18.6 | 19.4 |  | 19.4 | 19.1 |  | 19.2 | 19.1 |  |
| Hospital with supra-regional SU ‡ | 68.4 | 67.6 | 68.7 |  | 66.2 | 68.8 |  | 66.7 | 69.2 |  |
|  |  |  |  |  |  |  |  |  |  |  |
| **3 months post-stroke** |  |  |  |  |  |  |  |  |  |  |
| Barthel Index, % |  |  |  | <.0001 |  |  | <.0001 |  |  | <.0001 |
| Independent | 69.4 | 45.6 | 79.4 |  | 9.6 | 81.8 |  | 41.2 | 84.3 |  |
| Independent with little help | 25.3 | 41.6 | 18.5 |  | 63.8 | 17.3 |  | 45.0 | 14.9 |  |
| Dependent | 5.3 | 12.9 | 2.1 |  | 26.6 | 0.9 |  | 13.9 | 0.8 |  |
| Depressive Symptoms (PHQ-9), % § |  |  |  | <.0001 |  |  | <.0001 |  |  | <.0001 |
| None (0) | 15.2 | 9.0 | 18.7 |  | 3.5 | 17.6 |  | 8.6 | 19.5 |  |
| Minimal (1-4) | 35.9 | 12.7 | 40.5 |  | 8.9 | 41.4 |  | 26.9 | 41.8 |  |
| Mild (5-9) | 29.7 | 31.7 | 28.6 |  | 40.4 | 27.5 |  | 32.6 | 27.8 |  |
| Moderate (10-14) | 11.7 | 18.4 | 8.0 |  | 24.6 | 9.1 |  | 18.6 | 7.2 |  |
| Moderate Severe (15-19) | 5.1 | 10.1 | 2.3 |  | 16.5 | 2.8 |  | 9.9 | 2.0 |  |
| Severe (20-27) | 2.4 | 3.2 | 1.9 |  | 6.2 | 1.6 |  | 3.5 | 1.7 |  |
| Care by ambulant nursing service, % | 6.6 | 11.8 | 4.4 | <.0001 | 22.4 | 3.3 | <.0001 | 12.7 | 3.3 | <.0001 |

†Atrial fibrillation known and newly detected

‡ SU (Stroke Unit)

§ PHQ-9 (Patient Health Questionnaire) data collected between 07/2017 and 06/2019

Table II Results of the Caregiver Reaction Assessment – positive and negative experiences of FC

| N=1,044 | Strongly disagree, % | Disagree, % | Neither agree nor disagree, % | Agree, % | Strongly agree, % |
| --- | --- | --- | --- | --- | --- |
| **Impact on finances** |  |  |  |  |  |
| Caring for ___ has put a financial strain on the family. | 35.0 | 22.2 | 25.6 | 11.8 | 5.5 |
| It is difficult to pay for ___’s health needs and services. | 27.6 | 19.3 | 32.7 | 12.6 | 7.8 |
| *My financial resources are adequate to pay for things that are required for caregiving. | 18.9 | 15.4 | 27.4 | 19.0 | 19.4 |
| **Lack of family support** |  |  |  |  |  |
| Others have dumped caring for ___ onto me. | 65.3 | 17.6 | 10.1 | 4.4 | 2.6 |
| It is very difficult to get help from my family in taking care of ___. | 42.6 | 23.0 | 16.6 | 9.9 | 8.0 |
| Since caring for ___, I feel my family has abandoned me. | 60.0 | 22.9 | 11.9 | 3.1 | 2.1 |
| My family (brothers, sisters, children) left me alone to care for ___. | 47.7 | 25.0 | 16.6 | 6.5 | 4.3 |
| *My family works together at caring for ___. | 19.4 | 15.1 | 21.4 | 24.1 | 20.0 |
| **Impact on health** |  |  |  |  |  |
| Since caring for ___, it seems like I’m tired all of the time. | 34.1 | 20.8 | 22.6 | 14.9 | 7.6 |
| My health has gotten worse since I’ve been caring for. | 41.0 | 20.4 | 20.9 | 13.5 | 4.2 |
| *I have enough physical strength to care for. | 10.8 | 12.6 | 28.2 | 27.5 | 20.9 |
| *I am healthy enough to care for ___. | 8.2 | 7.6 | 25.5 | 36.3 | 22.4 |
| **Impact on daily schedule** |  |  |  |  |  |
| My activities are centered around care for ___. | 29.8 | 24.4 | 22.2 | 14.1 | 9.5 |
| I have to stop in the middle of work. | 40.7 | 19.2 | 17.7 | 18.1 | 4.2 |
| I visit family and friends less since I have been caring for ___. | 34.2 | 20.3 | 16.3 | 18.8 | 10.4 |
| I have eliminated things from my schedule since caring for___. | 26.9 | 18.9 | 17.5 | 26.1 | 10.7 |
| The constant interruptions make it difficult to find time for relaxation. | 25.1 | 21.1 | 24.8 | 20.1 | 9.0 |
| **Caregiver self-esteem** |  |  |  |  |  |
| I feel privileged to care for___. | 9.8 | 4.9 | 15.3 | 22.5 | 47.5 |
| I really want to care for___. | 9.5 | 4.1 | 13.5 | 25.4 | 47.6 |
| I will never be able to do enough caregiving to repay___. | 25.5 | 14.7 | 36.3 | 12.2 | 11.3 |
| Caring for ___ makes me feel good. | 9.1 | 5.5 | 26.8 | 30.1 | 28.5 |
| Caring for ___ is important for me. | 6.3 | 2.8 | 9.4 | 34.5 | 47.0 |
| I enjoy caring for___. | 8.3 | 7.2 | 34.1 | 27.0 | 23.4 |
| I resent having to take care of ___. | 68.3 | 19.1 | 9.9 | 1.7 | 1.0 |

Table III Patient characteristics including single items of the Barthel Index and family caregiver characteristics associated with self-rated burden of FC

| **Self rated burden** | ***β*** | **95% CI for *β*** | | **P value** |
| --- | --- | --- | --- | --- |
| **Patient characteristics** |  |  |  |  |
| Age patient | 0.30573 | 0.05911 | 0.55236 | 0.0153 |
| Sex (Man) | -3.65050 | -9.74151 | 2.44050 | 0.2392 |
| BI Mobility | -0.34186 | -1.27577 | 0.59205 | 0.4719 |
| BI Feeding | -1.77430 | -3.23483 | -0.31377 | 0.0174 |
| BI Bathing | -2.06962 | -3.40714 | -0.73211 | 0.0025 |
| BI Toilet use | 0.70918 | -0.56613 | 1.98449 | 0.2747 |
| BI Bladder | -0.97106 | -1.86714 | -0.07497 | 0.0338 |
| Depressive Symptoms | 1.15361 | 0.66699 | 1.64022 | <.0001 |
| Ambulant nursing service | 7.20489 | -1.14498 | 15.55476 | 0.0905 |
| **Caregiver characteristics** |  |  |  |  |
| Age | -3.75918 | -7.31315 | -0.20521 | 0.0382 |
| Sex (Man) | -0.86983 | -7.09102 | 5.35135 | 0.7834 |
| Hours of care/week | 0.12245 | 0.05187 | 0.19304 | 0.0007 |

Figure I Flow Chart


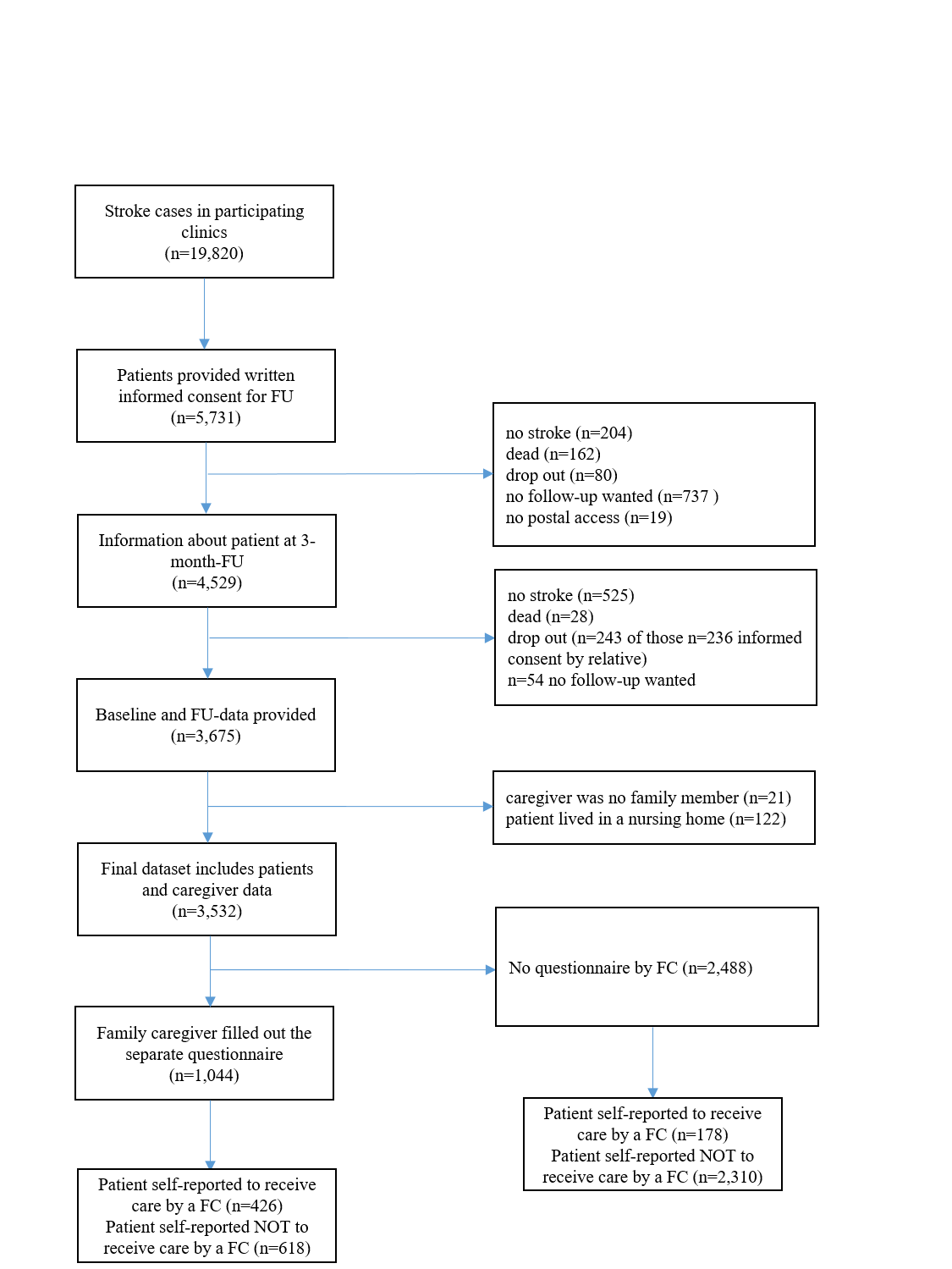

Supplement: Supplementary file 1 — Additional file 1. [file 12877_2022_2919_MOESM1_ESM.docx]
